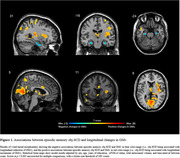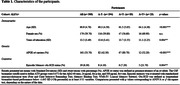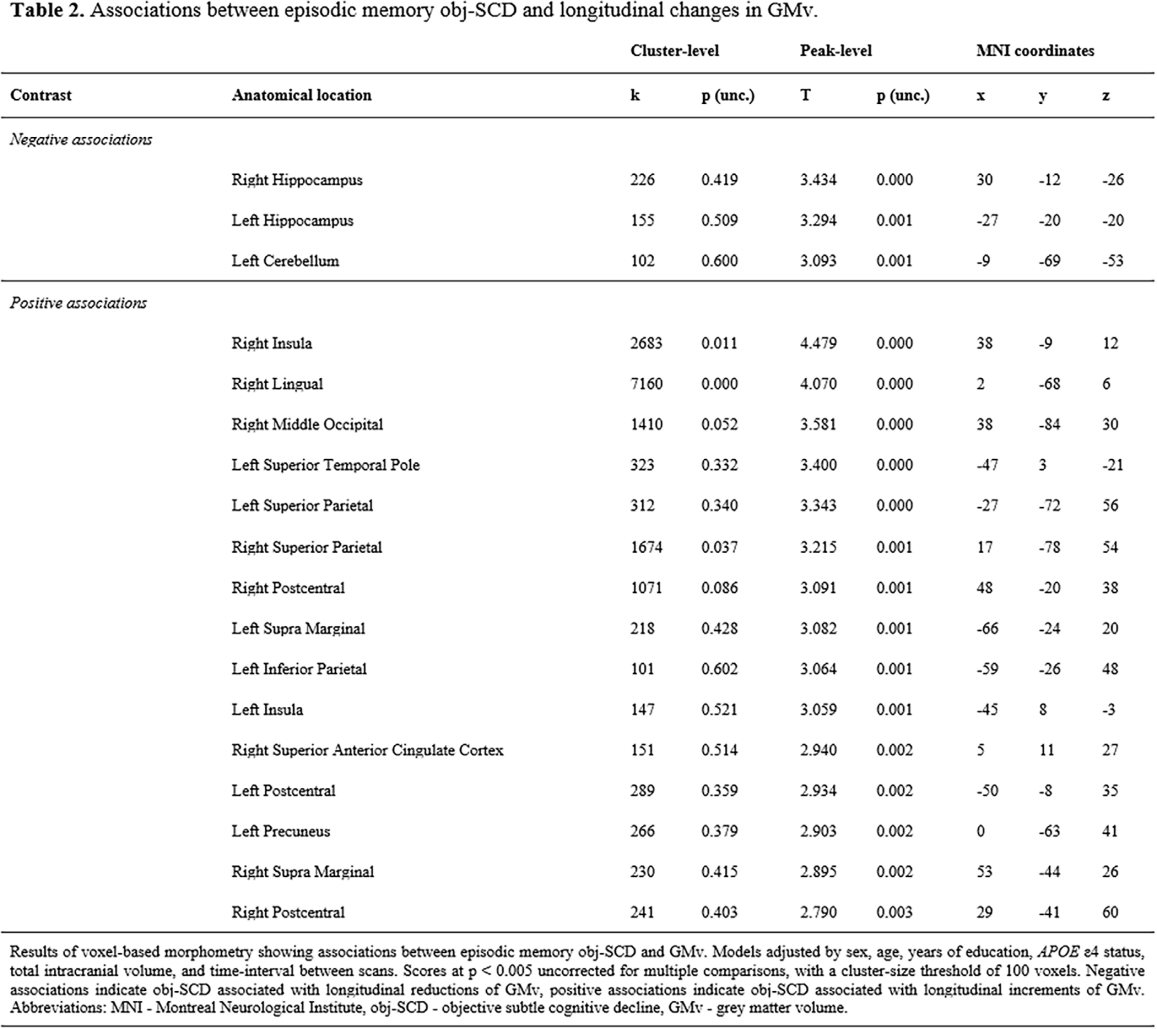# Sensitive methods to define subtle memory decline capture intra‐individual hippocampal atrophy and increased cortical volumes in preclinical Alzheimer’s

**DOI:** 10.1002/alz.085873

**Published:** 2025-01-03

**Authors:** David López‐Martos, Raffaele Cacciaglia, Marc Suárez‐Calvet, Marta Milà‐Alomà, Carolina Minguillon, Henrik Zetterberg, Kaj Blennow, Juan Domingo Gispert, Oriol Grau‐Rivera, Gonzalo Sánchez‐Benavides, for the ALFA study

**Affiliations:** ^1^ Barcelonaβeta Brain Research Center (BBRC), Pasqual Maragall Foundation, Barcelona Spain; ^2^ Hospital del Mar Research Institute (IMIM), Barcelona Spain; ^3^ Centro de Investigación Biomédica en Red de Fragilidad y Envejecimiento Saludable (CIBERFES), Madrid Spain; ^4^ Hospital del Mar Medical Research Institute (IMIM), Barcelona Spain; ^5^ Servei de Neurologia, Hospital del Mar, Barcelona Spain; ^6^ Centro de Investigación Biomédica en Red de Fragilidad y Envejecimiento Saludable (CIBERFES), Instituto de Salud Carlos III, Madrid Spain; ^7^ Department of Radiology and Biomedical Imaging, University of California, San Francisco, San Francisco, CA USA; ^8^ Department of Veterans Affairs Medical Center, Northern California Institute for Research and Education (NCIRE), San Francisco, CA USA; ^9^ Centro de Investigación Biomédica en Red de Fragilidad y Envejecimiento Saludable (CIBERFES), 28089, Madrid Spain; ^10^ Hong Kong Center for Neurodegenerative Diseases, Hong Kong China; ^11^ Wisconsin Alzheimer’s Disease Research Center, University of Wisconsin School of Medicine and Public Health, Madison, WI USA; ^12^ Clinical Neurochemistry Laboratory, Sahlgrenska University Hospital, Mölndal Sweden; ^13^ UCL Institute of Neurology, Queen Square, London United Kingdom; ^14^ Department of Psychiatry and Neurochemistry, Institute of Neuroscience and Physiology, the Sahlgrenska Academy at the University of Gothenburg, Mölndal Sweden; ^15^ UK Dementia Research Institute at UCL, London United Kingdom; ^16^ Department of Psychiatry and Neurochemistry, Institute of Neuroscience and Physiology, University of Gothenburg, Mölndal Sweden; ^17^ Hospital del Mar Research Institute, Barcelona, Barcelona Spain; ^18^ Centro de Investigación Biomédica en Red de Bioingeniería, Biomateriales y Nanomedicina (CIBER‐BBN), Madrid Spain; ^19^ Hospital del Mar Research Institute, Barcelona Spain; ^20^ Barcelonaβeta Brain Research Center (BBRC), Barcelona Spain

## Abstract

**Background:**

Objective Subtle Cognitive Decline (obj‐SCD) can be identified through standardized neuropsychological tests and may precede the development of Mild Cognitive Impairment (MCI). Nevertheless, current clinical and research criteria lack a standardized protocol for identifying obj‐SCD. This study introduces cutting‐edge sensitive methods to characterize obj‐SCD, defined through Alzheimer’s disease (AD) biomarker‐based longitudinal cognitive performance in episodic memory. Neurocognitive characterization of obj‐SCD is supported by Voxel‐Based Morphometry (VBM) analyses, examining longitudinal changes in Grey Matter volume (GMv) at the preclinical stage of the Alzheimer’s *continuum*.

**Method:**

Three hundred cognitively unimpaired (CU) individuals (mean age: 60, SD: 4.76) from the ALFA+ cohort study (three‐year follow‐up) were included. AT(N) profiles were defined at baseline with Cerebrospinal Fluid (CSF) biomarkers. AD biomarker‐based reliable change indices adjusted for practice effect (A‐T‐[N]‐ longitudinal performance as reference) were computed for the assessment of episodic memory (Free‐Cued Selective Reminding Test, Memory Binding Test, Wechsler Memory Scale‐IV). Considering the relationship between the number of neuropsychological measures and the base rate of impaired scores, obj‐SCD was defined as longitudinal biomarker‐based performance below ‐1.645 SD (<5^th^ percentile) in at least 3/11 variables. Magnetic Resonance Imaging scans were performed with 3T‐scanner and a high‐resolution 3D T1‐weighted sequence (voxel‐size: 0.75mm^3^). Intra‐individual changes in GMv were voxel‐wise computed using longitudinal scans, smoothed at 8mm^3^. The associations between obj‐SCD and GMv changes were analyzed with VBM linear regression models, selecting a voxel‐wise statistical threshold of *p*<0.005 with a cluster‐extent correction of 100 voxels.

**Result:**

According to the above‐defined criteria 19 (6.33%) participants exhibited episodic memory obj‐SCD, with significant differences considering AT profiles (Table‐1). Episodic memory obj‐SCD was associated with longitudinal reductions of GMv bilaterally in the hippocampus and the left cerebellum, as well as widespread increments of GMv involving AD‐vulnerable cortical regions (Table‐2, Figure‐1).

**Conclusion:**

Episodic memory obj‐SCD captured longitudinal changes in GMv indicative of AD‐progression (*i.e*., hippocampus). These results suggested that episodic memory obj‐SCD is a consistent marker of AD‐related impairment. Characterizing obj‐SCD enhances preclinical stage identification, with implications for advancing early detection and intervention strategies in Alzheimer’s disease, informing about an elevated risk of AD‐dementia in an otherwise CU population.